# Impact of the COVID-19 Pandemic on Online Obsessive-Compulsive Disorder Support Community Members: Survey Study

**DOI:** 10.2196/26715

**Published:** 2021-02-17

**Authors:** Benjamin Kaveladze, Katherine Chang, Jedidiah Siev, Stephen M Schueller

**Affiliations:** 1 Department of Psychological Science University of California, Irvine Irvine, CA United States; 2 Department of Psychology Swarthmore College Swarthmore, PA United States; 3 Department of Informatics University of California, Irvine Irvine, CA United States

**Keywords:** obsessive-compulsive disorder, COVID-19 pandemic, online support communities, mental health

## Abstract

**Background:**

People with obsessive-compulsive disorder (OCD) have faced unique challenges during the COVID-19 pandemic. Research from the first two months of the pandemic suggests that a small proportion of people with OCD experienced worsening in their OCD symptoms since the pandemic began, whereas the rest experienced either no change or an improvement in their symptoms. However, as society-level factors relating to the pandemic have evolved, the effects of the pandemic on people with OCD have likely changed as well, in complex and population-specific ways. Therefore, this study contributes to a growing body of knowledge on the impact of the COVID-19 pandemic on people and demonstrates how differences across studies might emerge when studying specific populations at specific timepoints.

**Objective:**

This study aimed to assess how members of online OCD support communities felt the COVID-19 pandemic had affected their OCD symptoms, around 3 months after the pandemic began.

**Methods:**

We recruited participants from online OCD support communities for our brief survey. Participants indicated how much they felt their OCD symptoms had changed since the pandemic began and how much they felt that having OCD was making it harder to deal with the pandemic.

**Results:**

We collected survey data from June through August 2020 and received a total of 196 responses, some of which were partial responses. Among the nonmissing data, 65.9% (108/164) of the participants were from the United States and 90.5% (152/168) had been subjected to a stay-at-home order. In all, 92.9% (182/196) of the participants said they experienced worsening of their OCD symptoms since the pandemic began, although the extent to which their symptoms worsened differed across dimensions of OCD; notably, symmetry and completeness symptoms were less likely to have worsened than others. Moreover, 95.5% (171/179) of the participants felt that having OCD made it difficult to deal with the pandemic.

**Conclusions:**

Our study of online OCD support community members found a much higher rate of OCD symptom worsening than did other studies on people with OCD conducted during the current COVID-19 pandemic. Factors such as quarantine length, location, overlapping society-level challenges, and differing measurement and sampling choices may help to explain this difference across studies.

## Introduction

The COVID-19 pandemic has led to population-level decreases in psychological well-being globally [1]. However, people with obsessive-compulsive disorder (OCD) may experience distinct pandemic-related stressors compared to the general population. Although many people with OCD might be particularly sensitive to pandemic-related stressors (eg, stressors related to contamination fears), social distancing measures might also provide a welcome respite from typical OCD triggers outside of one’s home. Research across various populations and timepoints is needed to learn how people with OCD have experienced the COVID-19 pandemic.

Online OCD support communities are a useful sample to consider as people seek support from online mental health communities for various reasons [2]. Online support communities may provide some unique advantages to traditional forms of support, including anonymity and use of access, which may enable greater self-disclosure and social support [3]. These communities may also be especially helpful in a pandemic context, wherein many in-person support sources may be lacking. Thus, exploring the impact the COVID-19 pandemic has had among individuals who participate in online support communities may be useful to understand the impact of the pandemic on help-seeking populations.

Several surveys among people with OCD were conducted between April and May 2020 in Israel [4], Italy [5], India [6], and Japan [7] to explore how OCD symptoms had changed since the onset of the pandemic. About 7%-37% of the participants in these surveys experienced worsening OCD symptoms since the COVID-19 pandemic began. Although our survey study used a methodology highly similar to these studies, we found a much higher rate of OCD symptom worsening. In this paper, we describe our survey of online OCD support community members and discuss possible reasons that contribute to the different patterns of results.

## Methods

We recruited the majority of our study participants from three anonymous online OCD peer support communities and the rest via posts on OCD-related social media pages. Participants indicated how much their symptoms in each of the four OCD symptom dimensions (“unacceptable thoughts,” “symmetry and completeness,” “responsibility for harm,” and “contamination”), as defined by the Dimensional Obsessive-Compulsive Scale [8], had changed since the pandemic began, with values ranging from 0 (much worse) to 6 (much better) that were transposed to –3 to 3, respectively, in our analyses for interpretability. Participants also indicated how much their OCD symptoms made living during the pandemic more difficult, with scores ranging from 0 (not at all more difficult) to 4 (much more difficult). The significance level for the statistical analysis was set at *P*<.05. Our preregistered study materials and analysis plan, as well as the survey data and R code used for analyses, are available online [9]. All available survey data has been reasonably de-identified by the research team and is being shared for research purposes with the consent of all study participants.

## Results

We conducted the survey and collected data between June 28 and August 10, 2020, and received 196 survey responses from individuals who stated they were professionally diagnosed with OCD, self-diagnosed, or suspected they had OCD. Not all participants answered all demographics questions. Most participants (n=163) were young (mean 24.77, SD 5.96 years), Caucasian (123/166, 74.1%), lived in the United States (108/164, 65.9%), and had their daily life affected by a stay-at-home order (152/168, 90.5%). In all, 71.4% (115/161) of the participants were female, 21.8% (35/161) were male, and 6.8% (11/161) reported their gender as other. We also excluded from the analyses an additional 134 respondents who exited the survey before completing the OCD measure.

**Figure 1:**
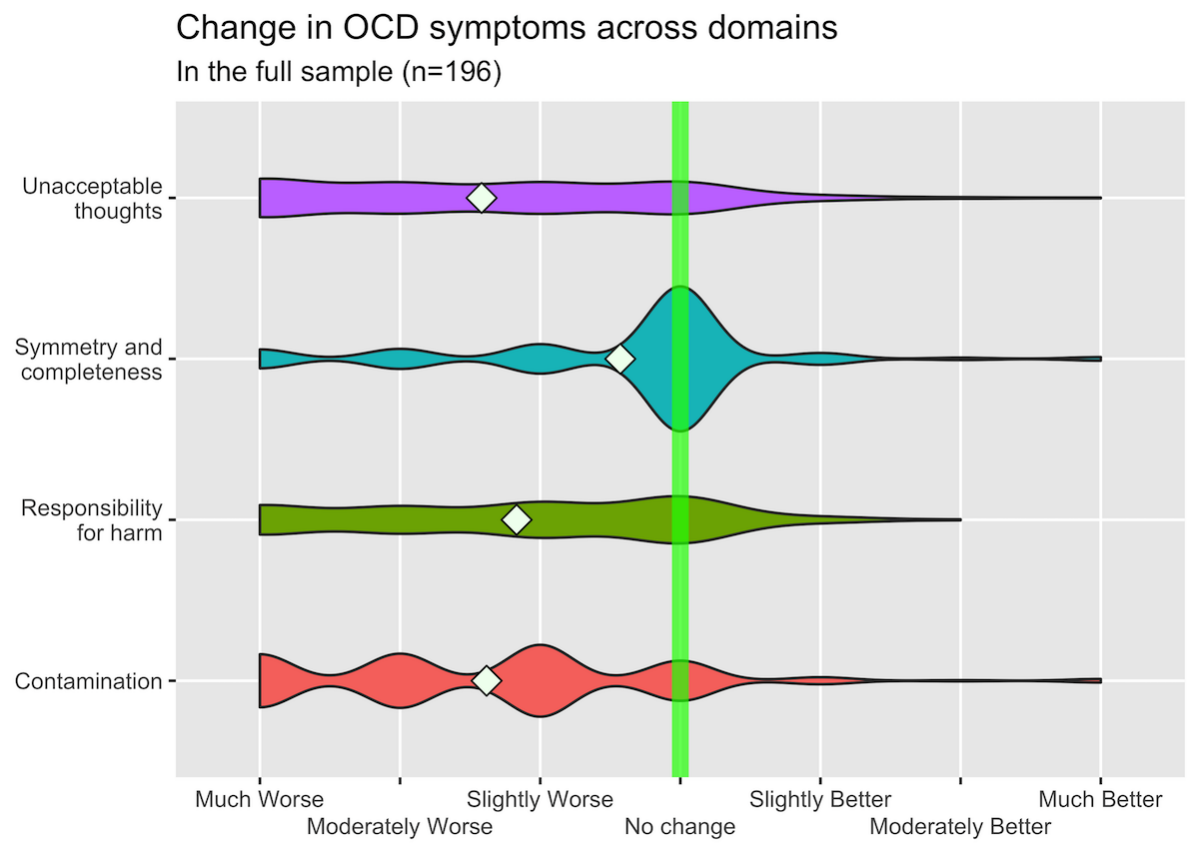
Changes in obsessive-compulsive disorder symptom severity across dimensions, as defined by the Dimensional Obsessive-Compulsive Scale. White diamonds correspond to sample means. Participants indicated the extents to which their symptoms across the four dimensions of obsessive-compulsive disorder (“unacceptable thoughts,” “symmetry and completeness,” “responsibility for harm,” and “contamination”) had changed since the onset of the COVID-19 pandemic, with scores ranging from −3 (much worse) to 3 (much better).

**Figure 2:**
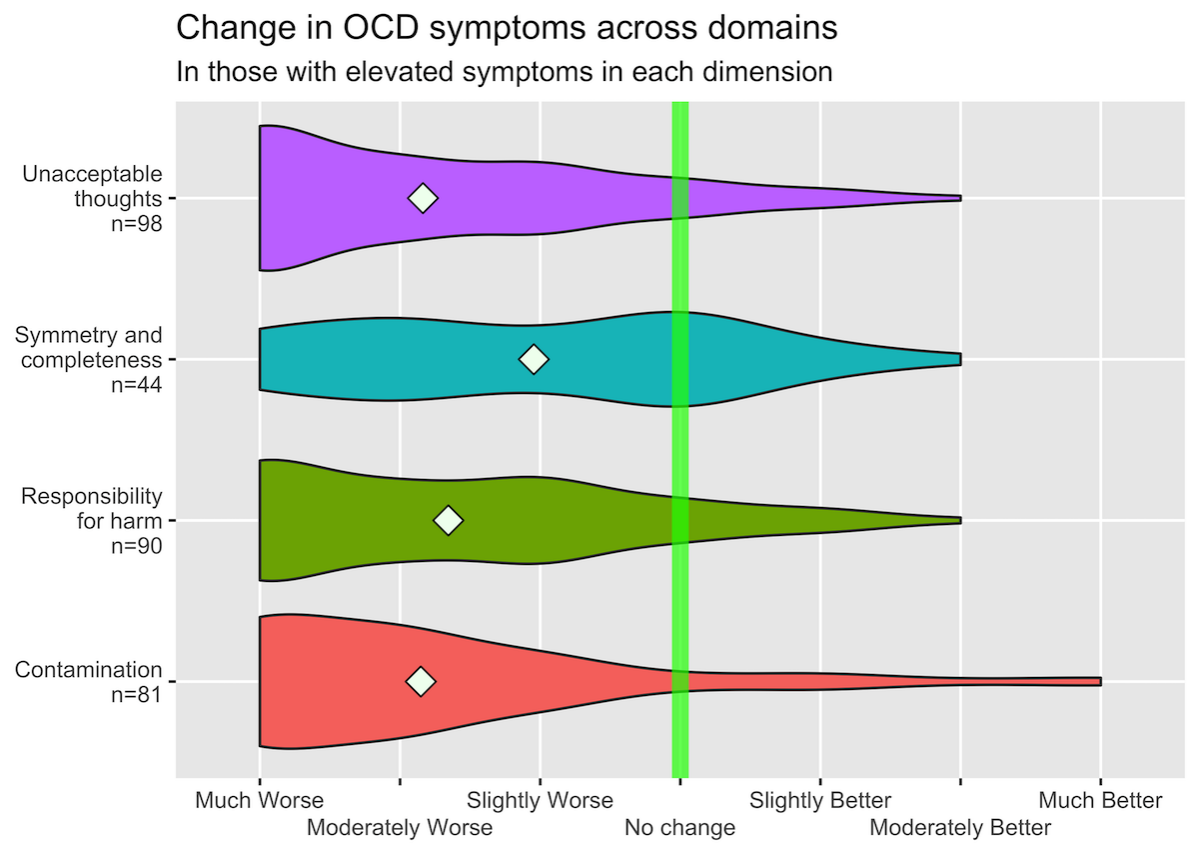
Changes in obsessive-compulsive disorder symptom severity across dimensions evaluated among only those participants with clinically elevated symptoms for a given dimension of obsessive-compulsive disorder, as defined by the Dimensional Obsessive-Compulsive Scale. We considered participants to have clinically elevated symptoms for a given symptom dimension if they scored an average score of 2 (range 0-4) across each item in that symptom dimension, which corresponds to moderate symptoms. White diamonds correspond to sample means. Participants indicated the extents to which their symptoms across the four dimensions of obsessive-compulsive disorder (“unacceptable thoughts,” “symmetry and completeness,” “responsibility for harm,” and “contamination”) had changed since the onset of the COVID-19 pandemic, with scores ranging from −3 (much worse) to 3 (much better).

We found that 92.9% (182/196) of the participants experienced worsening of their OCD symptoms since the COVID-19 pandemic began (mean −1.10, SD 0.80; *t*_195_=−19.35; *P*<.001; Cohen *d*=1.38). Notably, however, symmetry and completeness symptoms were considerably less worsened (Cohen *d*=0.38) than all other symptom dimensions of OCD (Cohen *d*>0.94), and the differences in symptom change between symmetry and exactness symptoms and other symptom dimensions were large and significant (*P*<.001; Figures 1 and 2). Moreover, 95.5% (171/179) of the participants felt that having OCD made it more difficult to deal with the pandemic, and 36.3% (65/179) of them indicated that having OCD made it much more difficult (mean 2.79, SD 1.16). When we included only those participants who stated that they had been professionally diagnosed with OCD in our analyses (n=142), the results were roughly the same (ie, <5% difference across samples in point estimates for OCD symptom worsening and OCD making it more difficult to deal with the pandemic).

## Discussion

### Principal Findings

Our results align with those of similar studies in some regards but differ importantly in other ways [4-7]. Similar to the findings reported by Littman et al [4], our results indicate that symmetry and completeness symptoms were less likely to worsen during the pandemic than symptom dimensions typically associated with harm. Overall, however, a much higher proportion of participants in this study reported worsening OCD severity during the pandemic, as compared with other studies. We believe that several factors may underlie the different results.

### Comparison With Prior Work

First, other studies were conducted in April and May 2020, that is, only a few weeks after quarantine measures were first instituted in their respective participants’ communities, whereas we conducted surveys between June 28 and August 10, 2020, that is, roughly 3 months after quarantine measures began for most of the US population. The mental health toll of pandemics may increase over time as anxiety, boredom, and frustration compound (as reviewed in [10]). Indeed, in the United States, symptoms of anxiety and depressive disorders in the population increased considerably more between April and June 2020 than they did between April and June 2019 [11].

Second, previous similar studies were conducted in Israel [4], Italy [5], India [6], and Japan [7], whereas most participants in our survey were from the United States. The mental health toll of COVID-19 is inextricable from location-specific factors; these include government decisions about the pandemic, media reports that might influence beliefs and reduce or increase psychological distress [12], and co-occurring collective challenges such as political unrest [13].

Third, online OCD support communities are likely disproportionately used by people seeking help because they are experiencing elevated symptoms. As a result, the communities we surveyed might have had higher proportions of people who felt their symptoms worsened since the COVID-19 pandemic began, compared to in-person populations (such as patients of an OCD clinic). Although Littman et al [4] also recruited a majority of their study sample from online OCD support groups, the communities they studied may have differed from the ones we studied with regard to the proportion of people seeking help in them.

### Limitations

We must note some limitations to our study. First, because we recruited our sample by posting advertisements on online OCD support communities and we do not know which members of these communities chose not to take the survey, our sample is not representative of any population of people with OCD. Second, our measure of OCD symptom change relied on retrospective self-report about the impact of the COVID-19 pandemic, asking participants how much their symptoms changed since the pandemic began; however, comparable studies either directly compared OCD measures before and after the pandemic using longitudinal data or did not mention the pandemic when asking respondents about their symptom change. As such, it might be most accurate to describe our findings as perceived worsening of symptoms. Despite these important limitations, we believe the extremely high rate of OCD symptom worsening among our survey participants remains noteworthy.

### Conclusions

Intersecting moderator variables such as quarantine length, location, overlapping society-level challenges, and public sentiment about quarantine measures complicate efforts to identify how the COVID-19 pandemic has affected people with OCD. Further, participants recruited from online support communities might be more likely to have sought help and experienced elevated symptoms at the time of data collection than typical in-person samples. The marked difference in the results between our study and other studies with similar goals and methodologies highlights the importance of considering these variables. As more studies on this topic are published, future works should use meta-analyses to investigate which sociocultural variables (eg, health care availability and trust in government) and researcher variables (eg, sampling and measurement choices) predict observed changes in OCD symptom severity during the COVID-19 pandemic.

## Abbreviations

OCD: obsessive-compulsive disorder
